# Pore size distributions and pore multifractal characteristics of medium and low-rank coals

**DOI:** 10.1038/s41598-020-79338-3

**Published:** 2020-12-18

**Authors:** Bin Sun, Qing Yang, Jie Zhu, Tangsha Shao, Yuhang Yang, Chenyu Hou, Guiyou Li

**Affiliations:** 1 Department of Coalbed Methane, Research Institute of Petroleum Exploration and Development, PetroChina, Beijing, 100083 China; 2 School of Mechanics and Civil Engineering, China University of Mining and Technology (Beijing), Beijing, 100083 China

**Keywords:** Coal, Characterization and analytical techniques, Petrology, Computational methods

## Abstract

It is of great significance to study the porosity and permeability properties of medium and low-rank coal. The porosity and permeability in confining stress experiments were used to simulate the porosity and permeability variations of coal samples under different depth conditions. The pore structure of Baoqing coal samples is greatly affected by the confining pressure, and the pores and micro cracks are more easily compressed. Based on the experimental data of mercury intrusion porosimetry (MIP) and nitrogen adsorption (NA), the pore size distributions (PSDs) of medium and low-rank coals were studied. High mercury intrusion pressure would lead to coal matrix compression. Therefore, the pore volume calculated by MIP data was corrected by NA data. The PSDs characteristics of Jixi (JX) coal and Baoqing (BQ) coal samples are obtained from the revised pore volume, and the dominant pores of medium and low-rank coals are obtained. The results show that JX coal has higher spatial heterogeneity, connectivity and pore autocorrelation. Micro fractures have an influence on the autocorrelation and heterogeneity of coal samples, especially for BQ coal samples.

## Introduction

With the change of energy structure, the role and status of unconventional natural gas in global oil and gas production has been strengthened^1^. Zou^2^ believes that to build a global green energy system, we need to vigorously improve the development of unconventional natural gas. To cope with climate and environmental change, it has become a consensus to accelerate the promotion of low-carbon energy. Natural gas and unconventional natural gas will significantly increase the proportion of main energy, among which coalbed methane (CBM) is an important part of unconventional natural gas. According to the latest prediction results, China is rich in coalbed methane resources, with the total reserve of 30.05 × 10^12^ m^3^, of which high rank accounts for 23%, medium rank accounts for 34%, and low-rank accounts for 43%^3,4^. The medium and low-rank coal resources account for 3/4 of the total coal resources. However, coal is a porous medium. The coal seam contains a rich pore network including pores and cracks. The characteristics of the pore structure affect the storage and migration of CBM^5,6^. The total amount of CBM resources in Jixi basin is 1874.87 × 10^8^ m^3^^7^, while that in Suibin depression is 1.87 × 10^11^ m^3^^8^. The two regions are rich in CBM resources, which are worth studying. The porosity and permeability of coal reservoir is one of the main factors affecting the success or failure of CBM resource development. The properties are in turn affected by the pore structure^9^.

Experimental methods such as mercury intrusion, specific surface test analysis, micro-CT scan, coal rock nuclear magnetic test, and small-angle X-ray scattering method are used to directly and quantitatively analyze the pores and micro cracks characteristics of porous media materials^10^. But the data of these experimental results need an effective method for summary analysis. Fractal geometry refers to irregular shape with self-similarity such as the length of coast. Fractal dimension is the main parameter to describe such self-similarity, which can quantitatively describe the complexity of a fractal geometry^11^. The pore structure of the porous material (soil, metal and coal) has self-similarity, so it can be regarded as the fractal geometry^12–14^, and the fractal dimension is used to describe the porous structure Muller^15^ studied the flow characteristics of sedimentary rocks with fractal method. Caniego et al.^16^ studied the pore size distributions (PSDs) characteristics of soil using singular fractal dimensions. Gauden^14^ first applied the fractal method to describe the pore structure of coal, and combined with the pore data obtained by experimental methods, quantitatively revealed the pore structure characteristics of coal. Based on the modified Frenkel–Halsey–Hill (FHH)^17^ and Neimark method^18^, Sun^19^ used fractal methods to study coal samples in shallow and deep coal seams to study the CBM mining process. Li et al.^20^ considered the compressibility of coal matrix, studied the pore structure characteristics of modified fine-grained coal by mercury intrusion method, and characterized it by surface fractal method. Li et al.^21^ combined the experimental data of mercury intrusion porosimetry (MIP), nitrogen adsorption (NA) and carbon dioxide adsorption (CA) to analyze the size distribution of tectonic deformed coal, and studied the structural characteristics of coal by using multiple singular spectrum method and generalized multifractal method, respectively. Lan^22^ established models for the process of mercury intrusion and extrusion isotherm curves of high rank coal and rock with both pores and micro-fractures, to explore the relationship between the models and pore connectivity. Li et al.^23^ investigated the pore characteristics of coal specimens with bursting proneness.

To explore the pore and micro cracks structure characteristics of low and medium-rank coal, the experiments of MIP and NA methods have been carried out in the coal samples from Dongshan Coal Mine in Jixi Basin and Baoqing Coal Mine in Suibin depression, China. Combined with the experiments of overburden porosity and permeability, the PSDs and pore fractal characteristics are investigated. And the effect of micro fractures on fractal dimension was also studied.

## Coal sample background

The two groups of coal blocks were collected from Dongshan Coal Mine No. 7 coal seam (mining depth 840 m) in Jixi Basin and Baoqing Coal Mine No. 10 coal seam (mining depth 16.44 m) in Suibin depression, respectively. The cylindrical coal samples with 25 mm-diameter and 50 mm-length were drilled from each block along the bedding plane for overburden porosity and permeability experiments. After the porosity and permeability test, the fragments of coal samples were collected and screened into small coal blocks with particle size less than 1 cm × 1 cm × 1 cm to MIP and NA experiments. In the process of coal block drilling, we collected and screened coal powder into 60–80 mesh, which was used for coal maturity test, macerals test and coal industry analysis. The mentioned two kinds of coal samples were abbreviated in JX and BQ, respectively.

Based on GB/T16773-2008 (China), industrial analyses were carried out with particle size less than 1 mm to judge the types of JX and BQ coal samples. The results were listed in Table 1. The *R*_0,max_ of JX and BQ samples are 0.84% and 0.40%, respectively, which means that JX coal sample is medium-ranked and BQ coal sample is low-ranked. The moisture content of JX coal sample is far less than that of BQ coal sample, while the fixed carbon content of JX coal sample is much larger than that of BQ coal sample, which also reveals that JX coal sample has a higher degree of metamorphism.

**Table 1:** Results of proximate analysis and maceral of different samples.

Sample name	Exinite content (%)	Vitrinite content (%)	Inertinite content (%)	*R*_*0,*max_ (%)	Volatile (%)	Moisture (%)	Ash yield (%)	Fixed carbon (%)
JX	32.60	44.35	23.05	0.84	31.45	1.34	9.97	57.79
BQ	29.60	65.60	4.80	0.40	53.47	41.33	7.78	22.73

The overburden porosity and permeability of JX and BQ coal samples were measured by AP608 overburden pressure porosimeter produced by Coretest company in the United States. An electronically controlled fluid injection pump has applied to adjust the overlying pressure. Transient pulse attenuation technique is employed to measure permeability. Based Boyle's law, porosity and pore volume of coal samples were evaluated. In order to determine permeability, helium is allowed to flow through a prepared rock sample with specific size, and the differential pressure and flow rate are measured. The inlet/outlet pressure the gas flow are measured with a manometer and a calibrated vent respectively. The test was conducted according to the standard SY/T 6385-1999 of China. The coal samples were dried and put into the overburden porosimeter (AP 608). The confining pressures were set as 3.5 MPa, 7 MPa, 14 MPa, 21 MPa and 28 MPa to simulate the confining stress on coal seam at different buried depths.

ASAP 2460 specific surface and porosity analyzer was employed in the low temperature liquid nitrogen adsorption test. The theoretical test range of pore size is 2–200 nm, and the specific surface area is 0.1–3500 m^2^/g. The test standard referred to the method of static nitrogen adsorption capacity for the determination of specific surface area and pore size distribution of rocks of CNPC (SY/6154-1995, China). The autopore IV 9500 mercury porosimeter was used in the MIP experiment, and the test standard was followed the determination of capillary pressure curve of rock (SY/TSP346-2012, China). The maximum pressure is 60,000 psi (413.79 MPa), and the pore diameter measurement range is 3 nm to 1000 μm. The MIP method could quantitatively obtain the pore distribution parameter information of pore size, pore diameter distribution and pore structure type.

## Results and data analyses

### Porosity and permeability under the confining stress results

**Figure 1:**
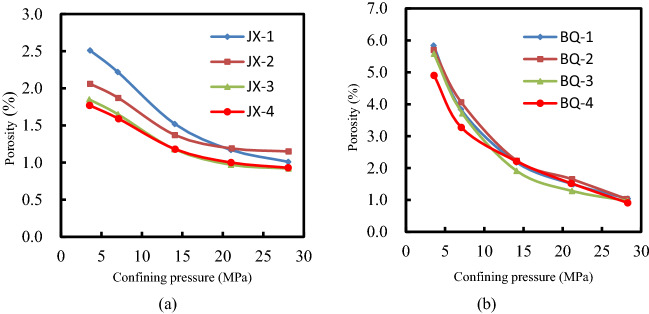
The relationship between porosity and net confining stress.

The confining porosity isotherms for coal samples are shown in Fig. 1. Figure 1 demonstrates big differences between the coal samples studied. With the accession of confining pressure, the average porosity of JX coal samples decreases from 2.15 to 1.02%, while that of BQ coal samples significantly decreases from 5.60 to 1.01%. And the higher confining stress is, the lower the porosity of coal. It is revealed that the effect of load on the pore compaction of coal sample is consistent with the research conclusion of Song et al.^24^. When the confining pressure is 21 MPa, the minimum porosity of JX coal sample is 0.97% (corresponding to JX-3 coal sample), and the average porosity is 1.11%. Meanwhile, the minimum porosity of BQ coal sample is 1.28% (corresponding to BQ-3 coal sample), and the average porosity is 1.50%.

**Figure 2:**
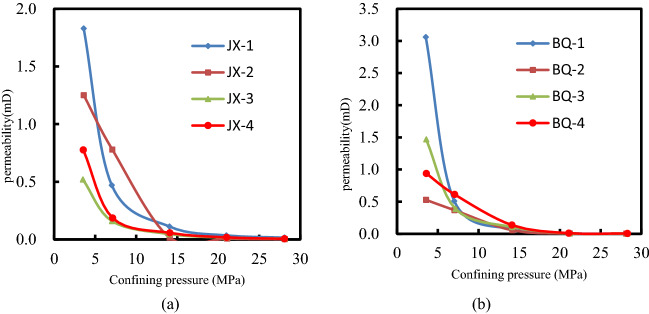
The relationship between permeability and net confining stress.

Figure 2 displays the confining permeability isotherms for JX and BQ coal samples. The confining pressure increases from 3.5 to 28 MPa, and the average permeabilities of JX coal samples decreases from 1.243 to 0.013 mD, and the permeability mean of BQ coal samples lessens from 1.364 to 0.003 mD; when the confining pressure is 21 MPa, the average permeability of JX and BQ coal samples is 0.034 mD and 0.006 mD, respectively. From the macroscopic phenomenon analysis, the pore fissures in coal are gradually compacted with the increase of effective stress, and the gas flow is blocked. With the increase of effective stress, the change trend of permeability of coal becomes smaller and smaller. From the microanalysis, it is concluded that with the application of load, the internal pores and fissures of coal keep closing, so that the gas passing through the test piece decreases.

Combining Figs. 1 and 2, it shows that both the porosity and permeability of the JX and BQ coal samples decrease significantly with the aggrandization of the confining pressures, and the variations of BQ coal samples are more obvious. This phenomenon reveals that BQ coal samples have more porosity and cracks content, and its closure degree is higher under external pressure. When the confining pressure increased from 3.5 to 7 MPa, the porosity and permeability of BQ coal samples decreased by 25% and 66%, respectively. It shows that the stress sensitivity of BQ coal samples is stronger, and the seepage pores are impacted by confining pressure more obviously.

### NA experimental results

The parameters of transition pores and micro pores obtained by NA experiment are listed in Table 2. Brunauer–Emmet–Teller method (BET) was used to acquire the specific surface area of JX and BQ coal samples (*S*_BET_). Barrett Joyner Halenda method (BJH) was used to obtain the total pore volume of coal samples (*V*_BJH_). In Table 2, the average specific surface area of JX coal samples is 0.249 m^2^/g, while the average specific surface area of BQ coal samples is 3.025 m^2^/g. In Table 2, the average median pore diameters (volume) of JX samples and BQ coal samples are 29.9 nm and 492.275 nm, respectively. And the median pore diameters of samples are dispersive because of coal heterogeneous characteristic. The content of transition pores and micro pores of JX coal sample is lower than that of BQ coal sample.

**Table 2:** The parameters obtained from the NA and MIP experiments.

Sample ID	Bulk density (g/cm^3^)	Skeletal density (g/cm^3^)	Porosity (%)	Parameters obtained from N_2_ injection				Parameters obtained from MIP experiments		
								Median pore diameters (nm)	Average pore diameters (nm)	Total pore area (m^2^/g)
				*S*_BET_	*V*_BJH_	*V*_trans_	*V*_micro_			
				m^2^/g	cm^3^/g	cm^3^/g	cm^3^/g			
JX-1	1.301	1.461	10.944	0.163	1.60 × 10^–4^	8.08 × 10^–5^	1.86 × 10^–5^	52.50	10.70	31.57
JX-2	1.341	1.424	5.821	0.300	4.39 × 10^–4^	2.50 × 10^–4^	3.22 × 10^–5^	10.10	9.20	18.86
JX-3	1.318	1.401	5.888	0.422	1.62 × 10^–3^	7.02 × 10^–4^	1.82 × 10^–4^	11.70	9.60	18.55
JX-4	1.267	1.404	9.719	0.110	4.85 × 10^–4^	2.18 × 10^–4^	9.17 × 10^–6^	45.30	11.30	27.10
Average	1.307	1.4223	8.093	0.249	6.09 × 10–4	3.13 × 10–4	6.05 × 10–5	29.9	10.2	24.02
BQ-1	0.984	1.422	30.794	2.753	1.18 × 10^–2^	6.16 × 10^–3^	1.68 × 10^–3^	358.50	66.10	18.95
BQ-2	0.896	1.432	37.435	3.169	1.46 × 10^–2^	7.44 × 10^–3^	1.92 × 10^–3^	597.00	94.70	17.65
BQ-3	0.935	1.421	34.260	3.090	1.43 × 10^–2^	7.30 × 10^–3^	1.71 × 10^–3^	464.10	72.10	20.34
BQ-4	0.897	1.355	33.825	3.088	1.39 × 10^–2^	7.33 × 10^–3^	1.72 × 10^–3^	549.50	122.70	12.31
Average	0.928	1.407	34.078	3.025	1.41 × 10^–2^	7.28 × 10^–3^	1.78 × 10^–3^	492.275	88.9	17.31

**Figure 3:**
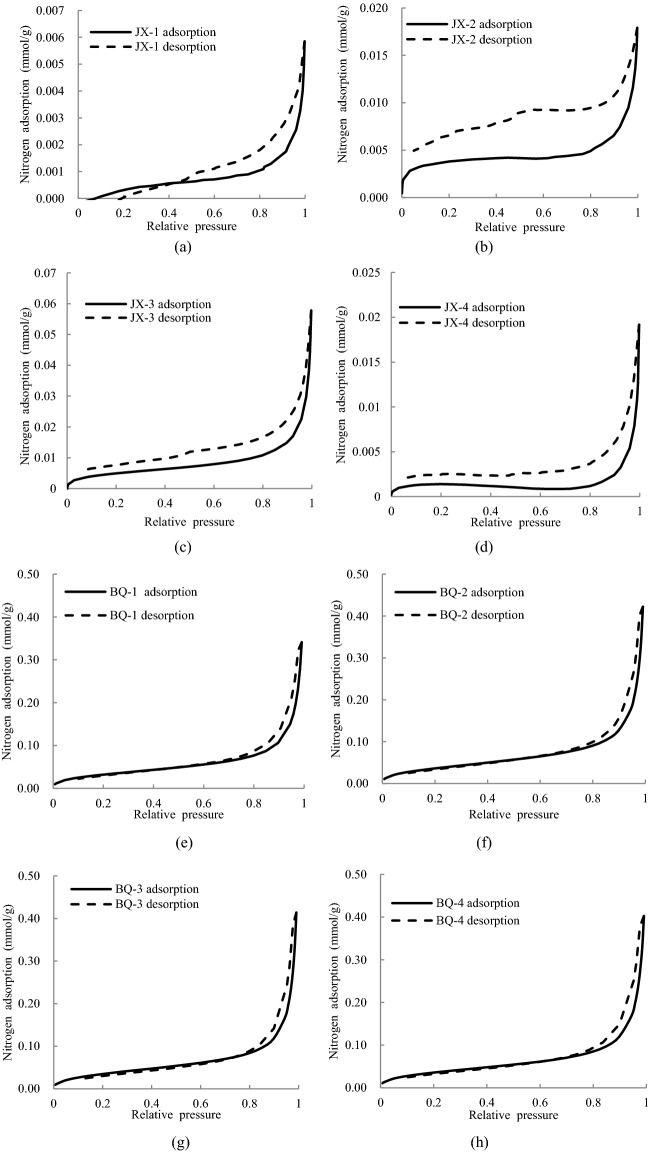
The isothermal adsorption/desorption curves of NA (p/p0 ≤ 1).

The relationship between nitrogen adsorption capacity and relative pressure (*p/p*_*0*_) for JX coal samples and BQ coal samples are shown in Fig. 3. As shown in Fig. 3, the nitrogen adsorption of BQ samples are nearly 10 times higher than that of JX samples, indicating that the micro pore volume content of BQ coal is much higher than that of JX coal. As the relative pressure rises, the nitrogen adsorption capacity of coal sample augments. When the relative pressure increases from 0.2 to 0.8, there is a positive correlation between the amount of nitrogen adsorption value and the relative pressure, which reveals that there is a certain amount of pore distribution in the pore size range corresponding to the relative pressure. As the relative pressure in the range of 0.8 to 1.0, the accumulative adsorption capacity of liquid nitrogen increases with a rush, and the adsorption isotherm curve was similar to the “exponential” form, which reveals that there is a large amount of pore distribution in the relevant pore size range.

When the nitrogen is desorbed, the desorption capacity of nitrogen cannot be equal to that of the relative pressure point. The area between desorption curve and adsorption curve is called hysteresis loop^25,26^. The width of hysteresis loop reveals the form of pore structure. JX-2 hysteresis loop is wider when the relative pressure is greater than 0.5, indicating that the closed pores at the sealed end are in a series of smaller pore diameters, while the open pores are in a larger pore diameter range. The adsorption isotherms of BQ samples have significant increase trends when the relative pressure is greater than 0.8, indicating that there are more small pores in BQ samples. There is almost no adsorption return line in the BQ adsorption isotherm, indicating that the BQ coal sample contains more air-tight pores closed at one end. The adsorption and desorption isothermal curves of all JX coal samples are not closed, as presented in Fig. 3. This is due to the volume expansion of the coal sample during the adsorption process or to the tiny pores in the coal sample adsorbed by the gas.

### MIP experimental results

**Figure 4:**
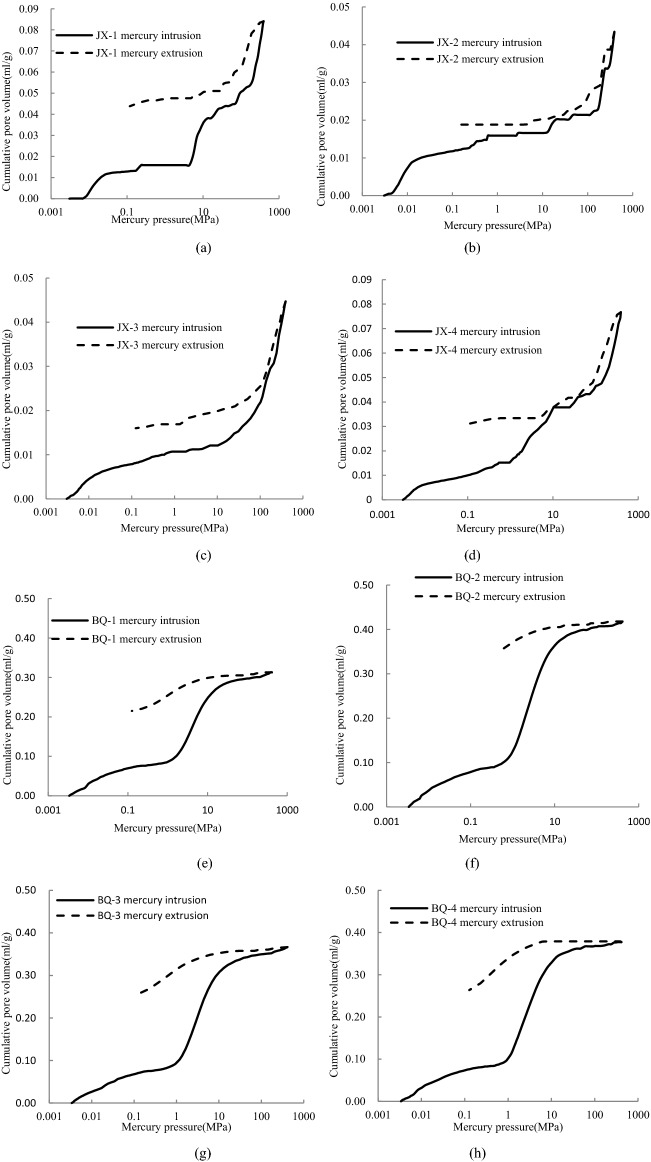
Mercury intrusion porosimetry curve.

Table 2 lists the parameters of MIP experiment including median pore diameters, average pore diameters and total pore area. The results show that the cumulative mercury intake of BQ coal is 5 times more than that of JX coal sample, and the average porosity, median pore size and average pore size of BQ coal sample are greater than that of JX coal sample, while the total pore area of BQ coal sample is smaller than that of JX coal sample. The intrusion and extrusion curves of MIP experiment are shown in Fig. 4, in which the x-axis is logarithmic. The intrusion isothermal curves of the JX samples are approximately "*L*" shape. As the increase of Hg pressure ranged from 0 to 70 MPa, there are a certain number of macro pores (including micro fracture) and meso pores, and JX-1and JX-4 sample have lots of meso pores. When Hg pressure heavier than 70 MPa, the Hg intrusion curves increases sharply. Meanwhile, the intrusion curve of the BQ sample is in the shape of "*S*", there are certain number of macro pores and minor micro pores, and a mass of meso pores.

Due to the existence of open pore, mercury can't retreat from the pore in time after the mercury pressure is reduced, which results in the hysteresis loop between the mercury intrusion curve and extrusion curve. As shown in Fig. 4, the hysteresis loops of the JX coal samples are narrow, and the hysteresis loops of the BQ coal samples are wide. This indicates that the transition pores and micro pores of the BQ coal samples contain more open pores, while the JX coal samples mainly contain semi-open pores.

### Pore size distributions

Coal is a porous medium, the coal matrix is compressed under a high mercury intrusion pressure (≥ 20 MPa). When the mercury pressure is greater than 400 MPa, the coal matrix is significantly compressed and the smaller pores are crushed. The pore volume is quite different from the actual pore volume^20,27^. The compressibility coefficient of the coal matrix is expressed by *K*_c_, and *K*_c_ is defined as^20^

1 $$ K_{c} = \frac{{dV_{c} }}{{V_{c} dp}} $$

where $\frac{{dV_{c} }}{dp}$ is the function of the coal matrix changing with pressure, and *V*_*c*_ is the coal matrix volume. Equation (1) ignores the compressibility of mercury.

Because coal contains numerous meso pores, micro pores, and super micro pores, even if the highest pressure provided by the experimental equipment is applied, mercury cannot enter some of them. *V*_*c*_ in the above equation also includes some unfilled pores. For compressible solid materials in the mercury intrusion experiment, we can define the observed change in the amount of mercury input *△V*_*obs*_:

2 $$ \Delta V_{obs} = \Delta V_{p} + \Delta V_{c} $$

*△V*_*p*_ is the pore filling amount; *△V*_*c*_ is the compression amount of the coal matrix volume.

**Figure 5:**
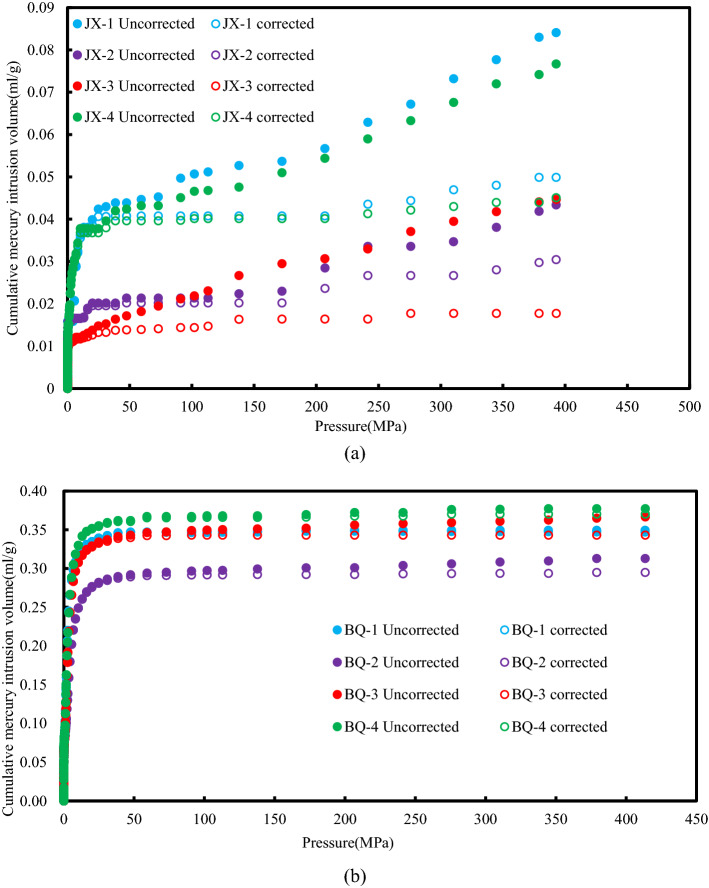
The cumulative mercury intrusion volume before and after correction.

Figure 5 shows a schematic diagram of the cumulative mercury intake of JX coal samples and BQ coal samples. When the mercury input pressure is high, the cumulative mercury intrusion curves of the two coal samples show a linear increase trend, and the fitted straight line of each mercury intrusion curve can be obtained by numerical fitting. The fitting coefficient R^2^ values of the fitted straight lines are all greater than 0.9, indicating that the volume of mercury entering the coal sample during the high pressure stage shows a linear increase in pressure. The same trend has been confirmed in previous studies^25^.

As shown in Fig. 5, we assume that *△V*_*obs*_* /△p* is a fixed value *β* in the high-voltage stage, so we can get it roughly by Eq. (3).

3 $$ \frac{{\Delta V_{c} }}{{\Delta V_{p} }} = \beta - \frac{{\mathop \sum \nolimits_{{R_{min} }}^{{R_{max} }} \Delta V_{p} }}{\Delta P} $$

The maximum pore diameter *R*_*max*_ and the minimum pore diameter *R*_*min*_ were obtained from the liquid nitrogen adsorption data of coal samples. The maximum pore diameter and minimum pore diameter are different for each coal sample. Assuming that $\frac{{\vartriangle V_{c} }}{{\vartriangle V_{p} }}$ is independent of pressure, by replacing $\frac{{dV_{c} }}{{V_{c} dp}}$ with $\frac{{\vartriangle V_{c} }}{{\vartriangle V_{p} }}$, the compressibility of the coal matrix can be obtained. That is:

4 $$ K_{c} = \left( {\beta - \frac{{\mathop \sum \nolimits_{{R_{min} }}^{{R_{max} }} \Delta V_{p} }}{\Delta P}} \right)/V_{c} $$

The compressibility of the coal matrix of coal samples was calculated. Therefore, the true volume of the coal-like coal matrix can be obtained from the true density and mass of the coal. According to the compressibility of the obtained coal matrix, the data of the mercury intrusion experiment was modified in this study to obtain the improved pore volume as Fig. 5 (consisting of the origin of the pores) shown. It can be seen from Fig. 5 and Table 3 that the compressibility of coal matrix with pore size less than 100 nm is obvious, which is consistent with the conclusion of Song et al.^28^. The corrected pore volume of BQ coal sample is still much larger than that of BQ coal sample, which shows that the coal sample after compressibility correction does not affect the structural characteristics of coal.

**Table 3:** Compressibility correction parameters.

Sample ID	*K*_c_ (× 10^−3^ MPa^−1^)	*β* (× 10^–5^)	*R*^2^	Cumulative pore volume (ml/g)	Corrected cumulative pore volume (ml/g)	Modification coefficient
JX-1	2.965	11.705	0.981	0.084	0.045	0.536
JX-2	1.161	2.926	0.945	0.043	0.020	0.465
JX-3	3.074	10.266	0.994	0.045	0.013	0.289
JX-4	2.514	10.074	0.989	0.077	0.037	0.481
BQ-1	9.036	25.345	0.991	0.313	0.290	0.927
BQ-2	8.925	21.950	0.907	0.418	0.399	0.955
BQ-3	7.342	25.220	0.913	0.367	0.339	0.924
BQ-4	9.971	19.919	0.914	0.377	0.363	0.963

Studies have shown that the compressibility of coal increases with decreasing coal rank (increasing micro pore volume). The average compressibility coefficient of JX coal samples is 2.523 × 10^–3^ MPa^−1^, while the average compressibility coefficient of BQ coal samples is 8.95 × 10^–3^ MPa^−1^. It is inferred that the micro pore volume content of the BQ coal sample is relatively large.

**Figure 6:**
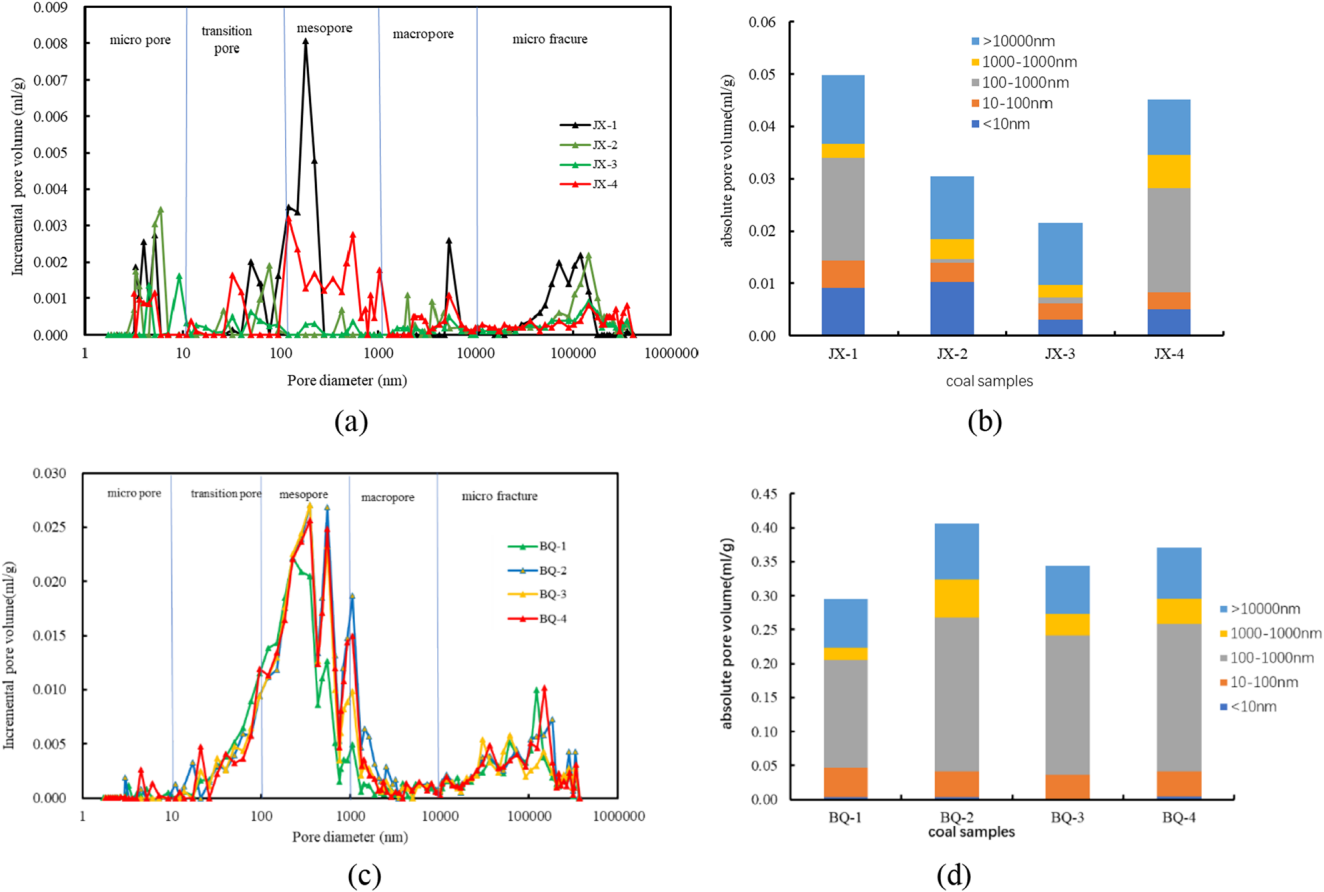
Modified pore size distributions and pore contents of different coal samples.

Figure 6 reveals the modified pore size distributions and pore contents of coal samples. The dominant pores of JX samples are almost microcracks, mesopores and micro pores, while the main pores of BQ samples are mesopores and microcracks. Generally speaking, pores with a diameter greater than 100 nm are called seepage pores^29^. The average porosity of BQ sample is 88.6%, which is higher than that of JX sample (69.1%). Combined with the pore volumes of the coal samples, and according to the pore characteristics, the pore structure of the BQ coal sample makes the gas easier to penetrate and flow.

## Discussions

### Multifractal analysis

Multifractal analysis is a quantitative and regular trend of geometrical irregularities in a certain range. It reveals the degree of heterogeneity and heterogeneity of material structure^26^. The data of the MIP experiments and the NA experiments, is used to calculate and analyze the multifractal dimensions of the materials. There are two equivalent mathematical methods to describe the characteristics of fractal geometry currently. Two multifractal mathematical methods, singular spectrum (*α*∼*f*(*α*))^30,31^ and generalized fractal dimension spectrum (*q*∼*D*(*q*)), were used to study characteristics of fractal geometry. The method of counting box dimension was used to analyze the corrected mercury intrusion volume.

The multifractal spectrum *f*(*α*) is a single peak convex function of *α*^32^. The two parameter values are obtained as follows^30^:

5 $$ \alpha \left( q \right) \propto \frac{{\mathop \sum \nolimits_{i = 1}^{N\left( \varepsilon \right)} \omega_{i} \left( {q,\varepsilon } \right){\text{lg}}\left[ {P_{i} \left( \varepsilon \right)} \right]}}{{{\text{lg}}\left( \varepsilon \right)}} $$

6 $$ f\left[ {\alpha \left( q \right)} \right] \propto \frac{{\mathop \sum \nolimits_{i = 1}^{N\left( \varepsilon \right)} \omega_{i} \left( {q,\varepsilon } \right)lg\left[ {\omega_{i} \left( {q,\varepsilon } \right)} \right]{ }}}{lg\left( \varepsilon \right)} $$

**Figure 7:**
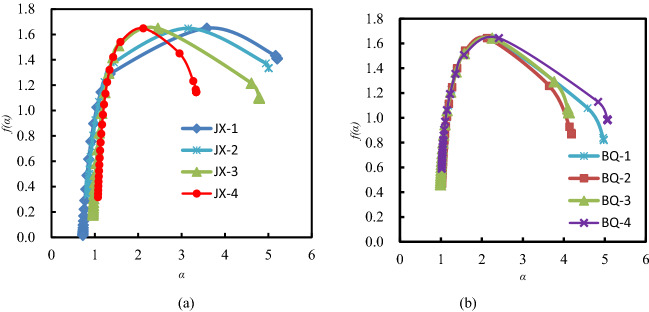
Multifractal singular spectrum of coal samples.

The multifractal singular spectrums for JX and BQ coal samples are illustrated in Fig. 7. The α ~ *f*(α) curves of all samples are convex parabola shape, that is, the PSDs of coal samples show multifractal behavior. The width of the *f*(α) spectrum reveals the complexity of the pore distribution of the coal sample. As *f*(α) spectrum width increases, the complexity of the pore size distribution increases. As shown in Fig. 7, the *f*(α) spectral width of the JX coal samples is wider than that of the BQ coal samples, indicating that the pore size distribution of the JX coal sample has higher spatial heterogeneity and complexity.

Using the same method as the multifractal singularity spectrum, the generalized fractal dimension $\left( {q\sim D_{q} } \right)$ was used to study the pore characteristics of coal samples. Similarly, in the generalized fractal measurement, using *P*_*i*_(*ε*)^*q*^ to highlight the local influence^30^,

7 $$ \mathop \sum \limits_{i = 1}^{N\left( \varepsilon \right)} P_{i} \left( \varepsilon \right)^{q} = \varepsilon^{{\left( {q - 1} \right)}} D_{q} $$

Then, *D*_*q*_ is expressed as^21,26^:

8 $$ D_{q} = \left\{ {\begin{array}{*{20}c} {\frac{1}{q - 1}\mathop {\lim }\limits_{\varepsilon \to 0} \frac{{log\mathop \sum \nolimits_{i = 1}^{N\left( \varepsilon \right)} P_{i} \left( \varepsilon \right)^{q} }}{\log \left( \varepsilon \right)} q \in \left[ { - 10,1} \right) \cup \left( {1,10} \right]} \\ {\mathop {\lim }\limits_{\varepsilon \to 0} \frac{{\mathop \sum \nolimits_{i = 1}^{N\left( \varepsilon \right)} P_{i} \left( {1,\varepsilon } \right)log\left[ {P_{i} \left( {1,\varepsilon } \right)} \right]}}{log\left( \varepsilon \right)} q = 1} \\ \end{array} } \right. $$

**Figure 8:**
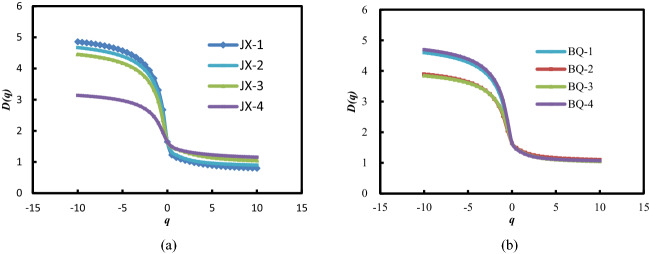
Generalized fractal dimension spectrum.

When *q* = 0, 1, 2, the meanings of dimension $$D_{q}$$ are capacity dimension, information dimension, and correlation dimension, respectively^33^. And $$D_{0} > D_{1} > D_{2}$$. When *q* > 0, the $$D_{q}$$ spectrum emphasizes areas with high porosity, and when* q* < 0, the $$D_{q} $$ spectrum emphasizes areas with low porosity^16^. The generalized fractal dimension spectrums are shown in Fig. 8.

Meanwhile, the Hurst exponent (*H* in short) is used to describe autocorrelation^34^, and its expression is^35^:

9 $$ H = (D_{2} + {1})/{2} $$

The value of *H* is also used in fractal analysis to characterize the pore autocorrelation of coal^36^. The average value of *H* value of JX and BQ coal samples is close to 1, indicating that the pore autocorrelation of the two coal samples is strong. After removing the micro fracture data, the *H* value of the two coal samples decreased to 1.026 and 1.065 respectively, that is, the autocorrelation of the two coal samples increased. It shows that the micro fracture enhances the autocorrelation of coal samples.

### The influence of micro fractures on fractal characteristics

After application of loading, the micro fractures in the coal seam are firstly affected, followed by the macropores and mesopores, and finally the transition pores and micro pores, and the larger the load, the more affected pores^37^.Wang^38^ studied that when the confining pressure of coal mass was kept constant at 3 MPa and the axial pressure increased from 0 to 30 MPa, the initial fracture compaction stage was experienced, and the original pore fracture in coal mass was closed under the action of external load, which was called the stage of linear elastic deformation. Based on this, the influence of micro fractures on the connectivity and heterogeneity of coal sample structure were studied with multi singular fractal dimension and multi generalized fractal dimension.

The singularity index α_0_ (*q* = 0) provides information on the concentration degree of pore volume distribution in coal samples. The higher the α_0_ value, the higher the heterogeneity of the pore volume distribution of the coal sample, and the more obvious the fluctuations^15^. As exhibited in Table 4, the average values of the multiple singular fractal index α_0_ of the JX and BQ coal samples are 2.826 and 2.259, respectively. The results show that the non-uniformity of pore distribution in BQ coal is lower than that in JX Coal.

**Table 4:** The calculated parameters of the multifractal singular spectrum and the generalized fractal dimension spectrum.

Sample number	*α*_0_	*α*_*q*+_	*α*_*q−*_	*α*_*0*_*–α*_*q*+_	*α*_*q−*_*–α*_*0*_	*α*_*q−*_*–α*_*q*+_	*H*	*D*_0_	*D*_1_	*D*_2_	*D*_10_	*D*_*−*10_	*D*_0_–*D*_10_	*D*_*−*10_–*D*_0_	*D*_*−*10_–*D*_10_
JX-1	3.581	0.724	5.203	2.857	1.622	4.479	1.026	1.649	1.146	1.052	0.803	4.859	0.846	3.210	4.056
JX-2	3.152	0.846	5.005	2.306	1.853	4.159	1.053	1.649	1.225	1.105	0.903	4.672	0.746	3.023	3.769
JX-3	2.454	0.959	4.791	1.495	2.337	3.832	1.161	1.648	1.418	1.321	1.046	4.456	0.602	2.808	3.410
JX-4	2.117	1.072	3.337	1.045	1.220	2.265	1.172	1.649	1.422	1.344	1.156	3.138	0.493	1.489	1.982
Average	2.826	0.900	4.584	1.926	1.758	3.684	1.103	1.649	1.303	1.206	0.977	4.281	0.672	2.633	3.304
BQ-1	2.237	1.046	4.974	1.191	2.737	3.928	1.133	1.643	1.382	1.265	1.095	4.597	0.548	2.954	3.502
BQ-2	2.130	1.034	4.191	1.096	2.061	3.157	1.142	1.643	1.400	1.283	1.097	3.889	0.546	2.246	2.792
BQ-3	2.254	0.991	4.125	1.263	1.871	3.134	1.123	1.643	1.371	1.246	1.050	3.845	0.593	2.202	2.795
BQ-4	2.415	1.020	5.066	1.395	2.651	4.046	1.116	1.643	1.355	1.232	1.068	4.695	0.575	3.052	3.627
Average	2.259	1.023	4.589	1.236	2.330	3.566	1.129	1.643	1.377	1.257	1.078	4.257	0.566	2.614	3.179

Not considering the micro fractures, the average value of singularity index α_0_ of JX and BQ coal samples is 2.676 and 2.400, respectively. It shows that the pore size distribution nonuniformity of JX coal samples after the removal of micro fractures decreases, while the pore size distribution nonuniformity of BQ coal samples increases. Microcracks have a significant impact on the pore size distribution nonuniformity.

With α_0_ as the bound, the left and right branches of the *f*(*α*) spectrums represent different variable information. The left branch *α*_*0*_ − *α*_*q*+_ (*q* > 0) corresponds to the high value of pore volume (area of dense pore volume distribution), and the right branch *α*_*q*_ − *α*_*0*_ (*q* < 0) corresponds to the low value of pore volume (Sparse area). The difference between the two parts *R*_d_ = ((*α*_*0*_ − *α*_*q*+_) − (*α*_*q*_ − *α*_*0*_)) indicates the degree of deviation of the fractal spectrum. If *R*_*d*_ > 0, the high value information has a significant effect on the pore space distribution; conversely, the low value information has a significant effect on the pore space distribution. Table 4 shows that the average values of *α*_*q*_ − *α*_*q*+_ for the coal samples from the Jixi mine is approximately equal to that of the BQ samples.

If the micro fracture data was removed, the R_d_ value of JX coal samples would increase from 0.168 to 0.306, while that of BQ coal samples would decrease from − 1.094 to − 2.753. It shows that after removing the micro fracture data, the influence of large pore volume ratio on the pore size distribution of JX coal sample is less. Meanwhile, BQ coal sample increases the influence of small pore volume ratio on the pore size distribution.

The generalized fractal parameters listed in Table 4. The length of *D*_*−10*_*–D*_*10*_*, D*_*0*_*–D*_*10*_ and *D*_*−10*_*–D*_*0*_ reveals the heterogeneity of the porosity. The larger the value of *D*_*−10*_*–D*_*10*_, the more uneven the pore size distribution. And the right side *D*_*0*_*–D*_*10*_ emphasizes high concentrations of porosity, while, the left side *D*_*−10*_*–D*_*0*_ emphasizes low concentrations of porosity^16,39^. For example, among all the coal samples, the *D*_*−*10_*–D*_10_ value of the JX-1 coal sample is the largest, indicating that the pore volume distribution heterogeneity of the JX-1 coal sample in different pore size intervals is the strongest. The average *D*_*−*10_*–D*_10_ value of the coal samples from the Jixi mine is bigger than that of the BQ samples by 0.125. In other words, the pore size distribution of JX coal is highly heterogeneous. The results are consistent with the analysis of the multiple singular fractal dimension spectrum. If the micro fracture data was not considered, the *D*_*−*10_*–D*_10_ value of JX coal samples would decrease slightly, while the *D*_*−*10_*–D*_10_ value of BQ coal samples would increase significantly, indicating that the micro fracture has a great impact on the degree of heterogeneity of BQ coal sample.

The closer information dimensions *D*_1_ is to capacity dimensions *D*_0_, the more uniform the porosity distribution is. In Table 4, the averages of the difference between *D*_1_ and *D*_0_ of JX and BQ coals are 0.346 and 0.266, respectively. That is, the pore size distribution of BQ coal is more even. Caniego stuck to that the smaller *D*_1_ is, the higher the heterogeneity is^16^. The average value of *D*_1_ of the JX coals is 1.303, which is less than that of the BQ coals. The result reveals that the size distribution of JX coal sample is more uneven. After removing the micro fracture data, the *D*_*0*_ and *D*_*1*_ values of JX coal sample and BQ coal sample decreased, which was caused by the decrease of pore data. The difference between *D*_0_ and *D*_1_ of the two coal samples increased, which increased the heterogeneity of pore size distribution.

## Conclusions

In this paper, the method of MIP and NA were used to study the PSDs of medium and low-rank coal samples. By applying different confining stress, the magnitudes of porosity and permeability variations for JX and BQ coal samples were obtained. Based on the multifractal theories, the pores fractal characteristics were discussed. We also studied the effect of micro fracture on the multifractal characteristics of coal samples. The main conclusions are as follows:

1. As the confining pressure increased, the porosity of the JX coal sample decreased from 2.15 to 1.02% and the permeability decreased from 1.243 to 0.013 mD, while the porosity of the BQ coal sample decreased from 5.60 to 1.01% and the permeability from 1.364 mD fell to 0.003 mD. The decline of BQ coal samples is sharper, especially the change of porosity. It shows that the pore structure of low-rank coal (BQ) is greatly affected by confining pressure, that is, the pore structure of BQ coal samples is greatly affected by the overburden depth, and the pores and micro cracks are more easily compressed. Therefore, the stress sensitivity of low-rank coal should be higher than that of medium rank coal.
2. The compressibility of coal matrix is not considered in the pore volume obtained by MIP experiment. When the mercury pressure is greater than 20 MPa, the real pore volume can be obtained by combining MIP and NA experimental data. The experimental results show that matrix compression coefficient of BQ coal sample is 3.55 times that of JX coal sample. After compressibility correction, the main pores of JX coal samples are macro pores (pore diameter ≥ 1000 nm) and meso pores (pore diameter100–1000 nm), and the proportion of pore volume is 40% and 23% respectively. The main pores of BQ coal samples are meso pores and macro pores, and the proportion of pore volume is 57% and 31%, respectively. The maturity of BQ coals and JX coals is different, so there is correlation between coal rank and dominant pore.
3. Two fractal methods, multifractal singular spectrum and generalized fractal dimension spectrum, are used to study the nonuniformity and connectivity of JX and BQ coal sample size distribution. JX coal sample of medium rank has more heterogeneity and connectivity than BQ coal sample of low rank, which indicates that JX coal sample is more conducive to the development of coalbed methane. Because the increasing of confining stress lead to the micro crack deformation and closure, the effects of the micro cracks has been evaluated in this work. It is indicated that the *D*_*−*10_–*D*_10_ value of JX coal sample will change little, while the *D*_*−*10_–*D*_10_ value of BQ coal sample is 1.656 times of the original. Therefore, the content of micro fractures may change the inhomogeneity of the two kinds of coal samples, especially for BQ coal samples.
